# Miscellaneous Items

**Published:** 1856-01

**Authors:** 


*Miscellaneous Items.*

The Virginia *Medical and Surgical Journal*, and *The Stethescope*, both formerly published at Richmond, Va., have been united, retaining the title of the former.

We observe an attempt is being made to establish, in the city of New York, an Hospital exclusively for the reception of "consumptive patients." If the object is to provide a comfortable place for that class of patients, who, in the advanced stage of phthisis, have no home or kind friends to protect them, after they become too feeble to protect themselves, then it is all very well. But if the plan embraces the idea of receiving and congregating together, within the walls of a Hospital, patients in all the stages of pulmonary tubercular disease, it is very questionable whether its evils would not overbalance its advantages.

*College of Physicians and Surgeons, of New York.*—This excellent medical college has just commenced the occupancy of the new and spacious college edifice, on the corner of 23d street and Fourth Avenue. We hope its future prosperity will be fully equal to its merits.

*Insanity, &c., in France.*—For every 100,000 inhabitants in France, there are 125 insane, 105 blind, 82 deaf and dumb, and 118 goitrous.

